# Preclinical evaluation and automated synthesis of [^89^Zr]ZrDFOSquaramide-girentuximab for diagnostic imaging of carbonic anhydrase IX positive tumours

**DOI:** 10.1186/s41181-024-00310-x

**Published:** 2024-11-26

**Authors:** Asif Noor, Emily R. McGowan, Jessica K. Van Zuylekom, Carleen Cullinane, Peter D. Roselt, Rodney J. Hicks, Michael P. Wheatcroft, Paul S. Donnelly

**Affiliations:** 1 School of Chemistry and Bio21 Molecular Science and Biotechnology Institute, University of Melbourne, Parkville, VIC 3010 Australia; 2 Research Division, Peter MacCallum Cancer Centre, Melbourne, VIC 3000 Australia; 3 Sir Peter MacCallum Department of Oncology, The University of Melbourne, Parkville, VIC Australia; 4 Melbourne Theranostic Innovation Centre, Level 8/14-20 Blackwood St, North Melbourne VIC, Melbourne, 3051 Australia; 5 Telix Pharmaceuticals Limited, Suite 401, 55 Flemington Road, North Melbourne, Melbourne, VIC 3051 Australia; 6 Department of Radiopharmaceutical Sciences, Cancer Imaging, Peter MacCallum Cancer Centre, Melbourne, VIC 3000 Australia

**Keywords:** Zirconium-89, Radiopharmaceuticals, DFO-squaramide, Automated synthesis, Zirconium-89 labelled antibodies, Girentuximab, Carbonic anhydrase IX

## Abstract

**Background:**

Carbonic Anhydrase IX (CAIX) is a zinc metalloenzyme that is over-expressed in many cancers making it a valid target for targeted diagnostic imaging with Positron Emission Tomography (PET). The monoclonal antibody girentuximab binds to CAIX and when radiolabelled with positron-emitting zirconium-89 can be used for diagnostic PET imaging of CAIX positive tumours.

**Results:**

Reaction of desferrioxamine squaramide ethyl ester with girentuximab allowed isolation of a conjugate with desferrioxamine squaramide (DFOSq) covalently attached to girentuximab through stable vinylogous amide linkages to give DFOSq-girentuximab. This conjugate was radiolabelled with zirconium-89 to give [^89^Zr]ZrDFOSq-girentuximab and the tumour uptake of the tracer was evaluated in CAIX positive HT29 tumour-bearing mice. Analysis of the PET images and biodistribution studies showed that the tracer displays high tumour uptake. An automated process for production of [^89^Zr]ZrDFOSq-girentuximab was developed, using [^89^Zr]ZrCl_4_ as a starting material that was also synthesized in an automated process. This automated process allows isolation of [^89^Zr]ZrDFOSq-girentuximab in radiochemical yields of 80–90% and in > 95% radiochemical purity.

**Conclusions:**

[^89^Zr]ZrDFOSq-girentuximab has high uptake in CAIX positive tumours. An automated procedure for the synthesis of [^89^Zr]ZrDFOSq-girentuximab using [^89^Zr]ZrCl_4_ as a starting material has been developed. This automated process could be readily adapted to other antibodies.

**Supplementary Information:**

The online version contains supplementary material available at 10.1186/s41181-024-00310-x.

## Background

The transcription factor Hypoxia Inducible Factor 1 (HIF-1) plays an important role in cellular adaptation to tumour hypoxia (Simone and Supuran 2010). Oxygen-dependent degradation of HIF-1 is initiated by interactions with the von Hippel–Lindau tumour suppressor protein (VHL). Inactivation of the VHL gene by mutation is a characteristic of clear cell Renal Cell Carcinoma (ccRCC) contributing to a hypoxic phenotype (Pastorekova and Gillies 2019). Among the cascade of hypoxia response genes, HIF-1 induces an increased expression of the gene that regulates expression of the zinc metalloenzyme, Carbonic Anhydrase IX (CAIX), an isoform that is capable of hydrating carbon dioxide to bicarbonate at a relatively low pH (Mahon et al. 2016). Consequently, CAIX is over-expressed in a significant proportion of ccRCC tumours as well as other cancers including breast, ovarian and colorectal (Pastorekova and Gillies 2019). The over-expression of CAIX in certain tumours and the fact there is relatively low expression in other tissues has seen this enzyme identified as a potential target for selective therapies.

**Fig. 1:**
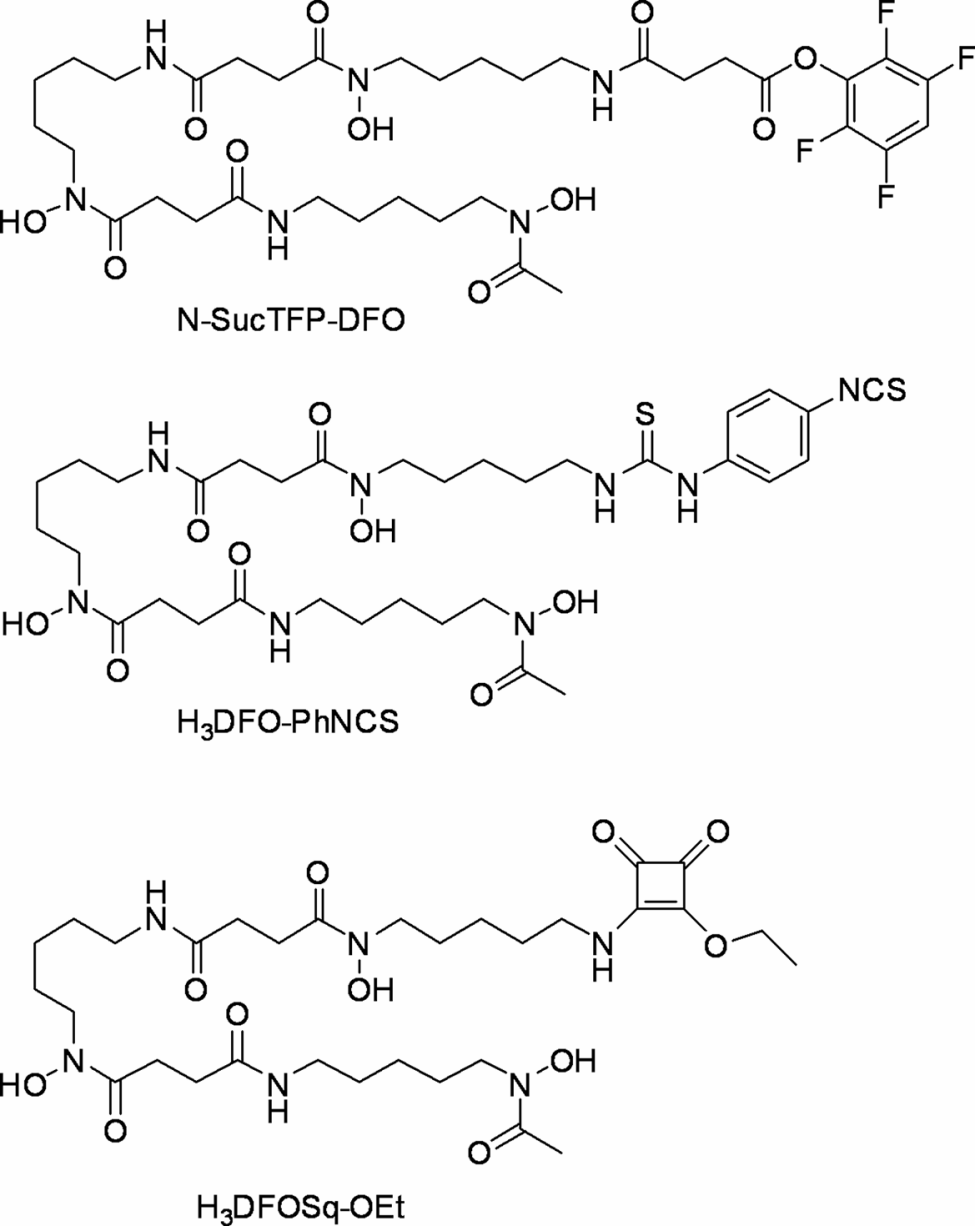
Chemical structure of N-SucTFP-DFO, DFO-PhNCS and DFOSq-OEt

The chimeric antibody girentuximab (also referred to as cG250) is selective for CAIX and binds to the extracellular proteoglycan-like domain. Diagnostic Positron Emission Tomography (PET) imaging with girentuximab radiolabelled with either positron-emitting iodine-124 or zirconium-89 has shown potential to be of use in identifying CAIX positive tumours (Cheal et al. 2014). Clinical studies with zirconium-89 labelled girentuximab attach the desferrioxamine chelator (DFO) to the antibody by reaction of desferrioxamine-N-succinyltetrafluorophenol ester (DFO-N-SucTFP, Fig. 1) with the antibody (Verhoeff et al. 2019, 2023; Merkx et al. 2021). The synthesis of DFO-N-SucTFP requires protection of the hydroxamic acid functional groups present in DFO by coordination to iron(III) which is then removed prior to conjugation and this adds several steps to the synthesis (Verel et al. 2003). An alternative approach to prepare DFO conjugates with antibodies is to react the antibodies with the isothiocyanate derivative, DFO − PhNCS (Fig. 1) (Cheal et al. 2014; Perk et al. 2010). Radiolabelled conjugates prepared using DFO − PhNCS give high-quality PET images (Vosjan et al. 2010; Deri et al. 2013), but when using DFO-PhNCS to modify antibodies, it is best to ensure a chelator: antibody ratio ≤ 1 to minimise aggregation. The reaction of DFO-PhNCS with antibodies attaches the chelator to the antibody through a thiourea linkage which is susceptible to decomposition by radiolysis (Vosjan et al. 2010; Vizier et al. 2024). 

An alternative bifunctional variant of DFO is desferrioxamine squaramide ethyl ester (DFOSq-OEt, Fig. 1), which can be readily prepared in a one-pot reaction between DFO mesylate and diethylsquarate without the need for protection of the hydroxamic acid functional groups (Rudd et al. 2016). DFOSq-OEt reacts selectively with the amine groups on lysine residues in antibodies to form stable aromatic vinylogous squaramide linkages to the antibody. In our experience, DFOSq-OEt can be used to prepare conjugates with chelator: antibody ratio of 3 − 4 without inducing aggregation or causing loss of biological function, and in contrast to DFO-PhNCS, the squaramide conjugates actually perform best at a chelator: antibody ratio of 3 − 4 (Rudd et al. 2016, 2021, 2024). Two DFOSq − antibody conjugates, [^89^Zr]ZrDFOSq − durvalumab and [^89^Zr]ZrDFOSq − DAB4, have been translated to human clinical trials (Hegi-Johnson et al. 2023; Liapis et al. 2021). 

In this work we report the synthesis of [^89^Zr]ZrDFOSq-girentuximab and evaluate its tumour uptake and biodistribution in CAIX positive HT29 xenograft mouse models. Encouraged by the promising pre-clinical PET images obtained using [^89^Zr]ZrDFO-N-suc-girentuximab and with the view of supporting potential multi-center clinical trials we then developed a consistent automated process for the synthesis of [^89^Zr]ZrDFOSq-girentuximab from [^89^Zr]ZrCl_4_. There are several advantages of automated procedures for the production of zirconium-89 labelled antibodies that include, minimizing radiation exposure during synthesis and facilitating the production of pure, sterile products in a reproducible fashion (Poot et al. 2019). The automated synthesis of [^89^Zr]ZrDFOSq-durvalumab, in an overall process yield of ~ 75%, using [^89^Zr][Zr(ox)_4_]^4-^ as a starting material has been published previously (Wichmann et al. 2023). In this work, we report the automated synthesis of [^89^Zr]ZrDFOSq-girentuximab, in an overall process yield of ~ 93%, using [^89^Zr]ZrCl_4_ as a starting material. The automated syntheses of the small molecule conjugates of DFOSq, [^89^Zr]ZrDFOSq-octreotate and [^89^Zr]ZrDFOSq-bisPhPSMA have also been published previously (Noor et al. 2024). 

## Results

### Synthesis of DFOSq-girentuximab

**Fig. 2:**
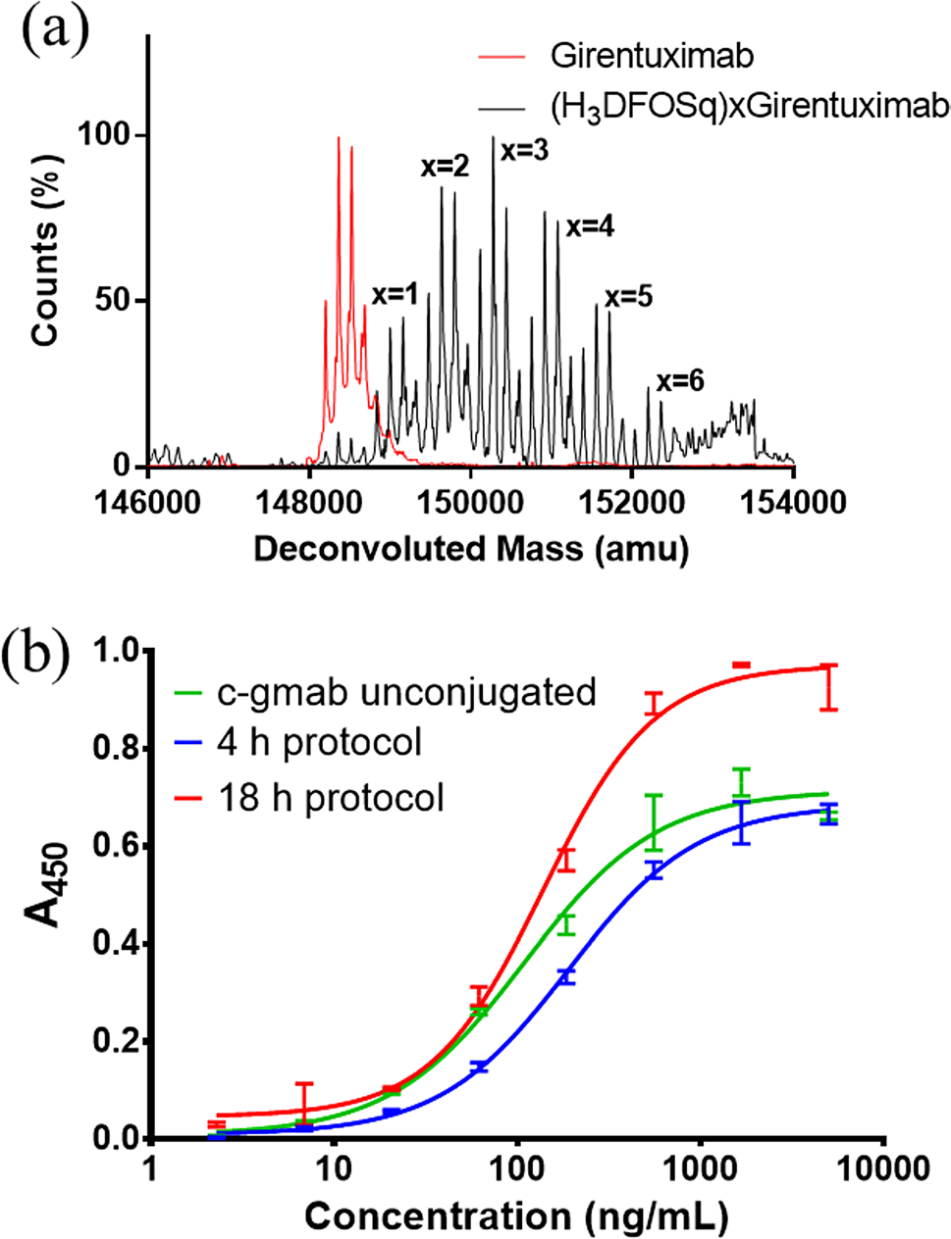
(**a**) Deconvoluted ESI-MS of DFOSq-girentuximab, (**b**) Representative ELISA curves for girentuximab and DFOSq-girentuximab

Girentuximab was reacted with increasing amounts of DFOSq-OEt to establish the optimal stoichiometry to produce conjugates where the average chelator: antibody ratio was 3–4. The antibody was reacted with 20, 40, 60 and 80 equivalents of DFOSq-OEt at a concentration of 1 mg/mL at room temperature for 18 h (Table S1). Any unreacted DFOSq-OEt was removed by spin filtration with size exclusion filters and the reaction mixture was analysed by intact protein electrospray mass spectrometry (ESI-MS). The deconvoluted ESI-MS spectrum of the product obtained using 40 equivalents of DFOSq-OEt reveals close to quantitative conversion of antibody to DFOSq conjugated products with an average of 3.3 chelators to antibody (Fig. 2a). The binding of DFOSq-girentuximab to CAIX was confirmed by an Enzyme Linked Immunosorbent (EC_50_ = 137 ± 1.0 ng/mL compared to girentuximab EC_50_ = 108 ± 1.1 ng/mL). It is possible to reduce the reaction time of the conjugation to 4 h using higher concentration of antibody (6 mg/mL) with DFOSq (40 equivalents) and mild warming (37 ^o^C) although this resulted in a DFOSq-girentuximab conjugate of with a higher EC_50_ (189 ± 1.0 ng/mL).

### Synthesis of [^89^Zr]ZrDFOSq-girentuximab

**Fig. 3:**
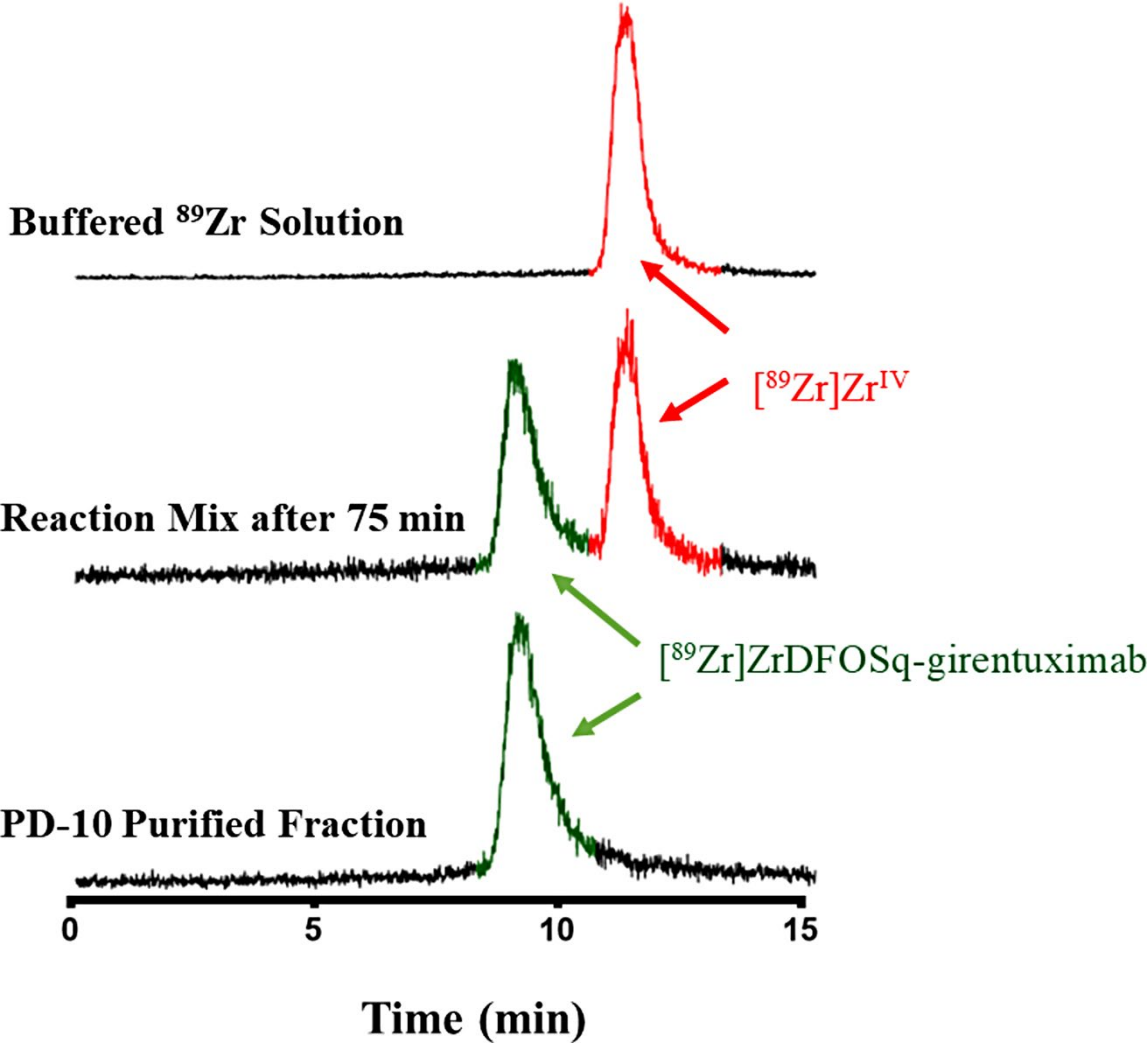
Radioactive size exclusion chromatograms monitoring the synthesis, purification and characterisation of [^89^Zr]ZrDFOSq-girentuximab

A mixture of [^89^Zr]Zr^IV^ in oxalic acid (1 M) was neutralized with Na_2_CO_3_ (1 M) and then added to HEPES buffer (0.25 pH 6–7) (HEPES = 4-(2-hydroxyethyl)-1-piperazineethanesulfonic acid). DFOSq-girentuximab was added to the mixture of [^89^Zr]Zr^IV^ in HEPES buffer at a ratio of 2 µg/MBq at room temperature. Analysis of the reaction mixture by size exclusion chromatography showed > 50% radiochemical yield after 75 min with no evidence of aggregation (Fig. 3). Purification using PD-10 size exclusion column provided [^89^Zr]ZrDFOSq-girentuximab in 55% radiochemical yield and > 95% radiochemical purity (500 MBq/mg). [^89^Zr]ZrDFOSq-girentuximab is stable in human serum, with no evidence of either dissociation of the [^89^Zr]Zr^IV^ or cleavage of the squaramide functional group linking the chelator to the antibody, when incubated in human serum for 48 h at 37 ^o^C (Figure S6).

### PET-CT imaging and biodistribution in HT29 tumour-bearing mice

**Fig. 4:**
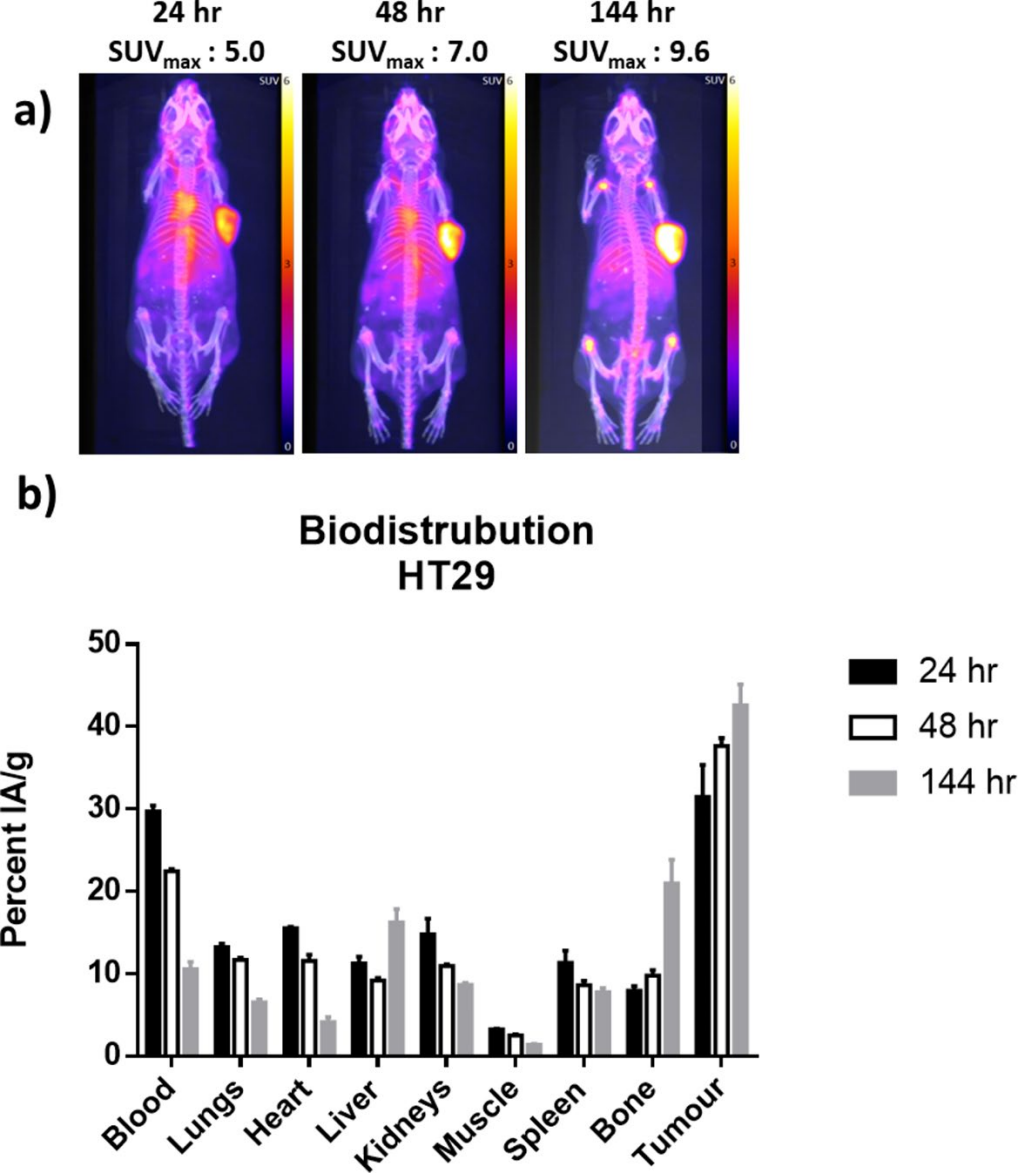
(**a**) Representative whole-body PET/Computed Tomography maximum intensity projection images of tumour-bearing mice (HT29) at 24, 48 and 144 h post injection of [^89^Zr]ZrDFOSq-girentuximab. (**b**) Ex-vivo biodistribution (tracer uptake is expressed as percent injected activity/gram tissue. Data represents mean ± SEM, *n* = 3)

[^89^Zr]ZrDFOSq-girentuximab was administered to HT29 tumour-bearing BALB/c mice (a high CA-IX expressing human colon carcinoma cell line)(Carlin et al. 2010; Mahalingam et al. 2018) and PET images were acquired at 24, 48 to 144 h post injection (Fig. 4a). At each time point there is very high tumour uptake and low background (SUV_max_ of 9.6 at 144 h post injection where SUV_max_ = Standardized Uptake Value = radioactivity in a tissue/injected activity/body weight). The retention in the blood pool, evident at both 24 h and 48 h post injection, is consistent with the relatively slow clearance of IgG antibodies due to their high molecular weight (~ 150 kDa) and Fc-mediated interactions (Fc = fragment crystallizable). An increase in bone uptake is evident in the images acquired at 144 h post injection particularly in the epiphysis of the long bones which can be partially attributed to anomalous biodistribution associated with Fc-mediated interactions in the immune deficient mouse model used (BALB/c nude) (Rudd et al. 2021, 2024; Sharma et al. 2018). The high tumour uptake of [^89^Zr]ZrDFOSq-girentuximab was confirmed by a biodistribution study where the amount of radioactivity that accumulated in the tumour and other organs was quantified at 24, 48 and 144 h post injection. The tumour uptake at 144 h post injection was 42.6 ± 2.6 IA/g (IA/g = injected dose per gram of tissue). The tumour uptake of [^89^Zr]ZrDFOSq-girentuximab was lower in low CAIX expressing MDA-MB-231 tumour-bearing mice (Figure S2, S3) (Mahalingam et al. 2018). 

### Automated synthesis of [89Zr]ZrDFOSq-girentuximab

**Fig. 5:**
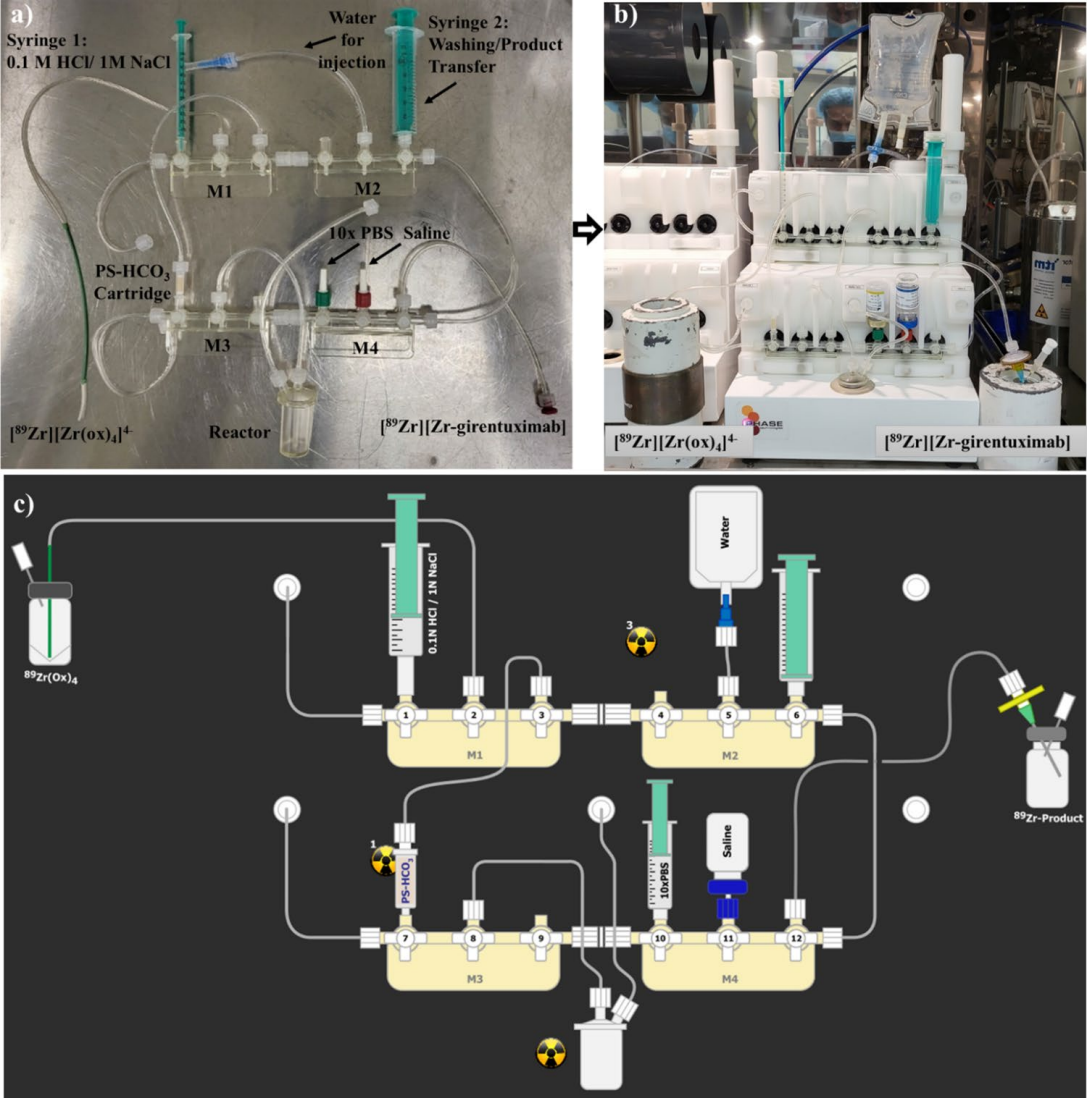
(**a**) A MultiSyn zirconium-89 disposable cassette. (**b**) Zirconium-89 disposable cassette on an iPhase Multisyn for the production of [^89^Zr]ZrDFOSq-girentuximab. (**c**) Schematic representation of the automated process

The promising imaging results obtained with [^89^Zr]ZrDFOSq-girentuximab encouraged us to develop an automated production process to potentially support future clinical studies. [^89^Zr]ZrCl_4_ was prepared using an automated process we reported previously (Noor et al. 2024). Briefly, a modified commercially available disposable cassette was configured and mounted to a commercially available iPhase synthesiser (Fig. 5a-b). A cartridge containing a strong anion exchange resin, a polystyrene-divinylbenzene polymer in the bicarbonate form (PS-HCO_3_ SAX cartridge, Huayi Isotopes), was used to trap [^89^Zr][Zr(ox)_4_]^4-^ (150–1000 MBq) on the cartridge (from [^89^Zr]Zr^IV^ mixtures in either 1 M or 0.05 M oxalic acid). Elution with a mixture of 0.1 M HCl (0.1 M) in aqueous NaCl (1 M) allowed isolation of [^89^Zr]ZrCl_4_ with in > 90% recovered yield. The [^89^Zr]ZrCl_4_ was directly eluted into a reaction vessel containing the DFOSq-girentuximab conjugate in aqueous sodium acetate (0.25 M) (Noor et al. 2024). The transfers of liquids were controlled through remote control software by opening and closing gas pressure/vacuum valves or by using the syringe drivers. This mildly acidic mixture of HCl (0.1 M) in aqueous NaCl (1 M) is readily neutralized by the aqueous sodium acetate present in the reaction vessel to give a mixture with a pH ~ 5–6. This mixture (pH 5–6) is suitable for radiolabelling with [^89^Zr]Zr^IV^ and alleviates the need to add a stronger base (sodium carbonate) which is required when [^89^Zr][Zr(ox)_4_]^4-^ in oxalic acid (1 M) is used as a starting material. After 30 min the reaction mixture was diluted with phosphate buffered saline (1 mL) using a syringe attached at position 10 in the manifold followed by addition of saline (0.9% w/v, 10 mL) from position 11 (Fig. 5). Following sterile filtration, the final product was collected in evacuated vials (20 mL), with a total volume of ~ 13 mL as a clear colourless solution with no visible particles and a pH of 6–7. Radiochemical yields ranging between 80 and 90% were obtained based on [^89^Zr][Zr(ox)_4_]^4-^ with a radiochemical purity of > 95% as determined by radio-HPLC and radio-TLC (Figure S4-5).

This automated process produced similar yields and radiochemical purity when two different commercial sources of [^89^Zr][Zr(ox)_4_]^4−^ were used that had different concentrations of oxalic acid (1 M or 0.05 M) and when the [^89^Zr][Zr(ox)_4_]^4−^ used within 1 day of production as well with [^89^Zr][Zr(ox)_4_]^4−^ that was 3 and 10 days post-production (Table 1). Similar radiochemical yields were also obtained with chimeric girentuximab (cGmAB) and a newly available humanized version of girentuximab (hGmAB) (Table 1). The procedures described here produced similar results with radioactivity ranging from 150 MBq to 1.03 GBq. To test the reproducibility of the process seven independent syntheses were performed and assessed by four parameters: Radiochemical purity, radiochemical yield, visual appearance and pH. Each independent synthesis resulted in > 95% radiochemical purity with radiochemical yields of 80–93%. Samples of [^89^Zr]ZrDFOSq-girentuximab (886 MBq) synthesized by this automated process are stable for at least 6 days when stored in the formulation buffer at room temperature (Figure S7).

**Table 1:** Automated synthesis of [^89^Zr]ZrDFOSq-girentuximab (hGmAB = humanized girentuximab, cGmAb = chimeric grientuximab) with different commercial sources of radioactivity, using radioactivity a differing number of days after production of the radionuclide and different amounts of radioactivity

	Variables				QC Specification			
	Zr-89-source (oxalic acid conc.)	GmAb Type	# of days after production of [^89^Zr]Zr	Scale MBq	RCP (iTLC)	Appearance	pH	RCY
1	Austin (0.05 M)	hGmAb	3 d	200	99%	clear, colorless	6–7	93%
2	Austin (0.05 M)	hGmAb	2 d	230	98%	clear, colorless	6–7	83%
3	Austin (0.05 M)	hGmAb	2 d	250	99%	clear, colorless	6–7	81%
4	Austin (0.05 M)	cGmAb	1 d	415	98%	clear, colorless	6–7	80%
5	Austin (0.05 M)	hGmAb	2 d	322	99%	clear, colorless	6–7	81%
6	Perkin Elmer (1 M)	hGmAb	10 d	180	96%	clear, colorless	6–7	80%
7	Austin (0.05 M)	hGmAb	1 d	1030	99%	clear, colorless	6–7	89%

## Discussion

The reactions of DFOSq-OEt with girentuximab allows isolation of DFOSq-girentuximab with an average chelator to antibody of 3:1. The new conjugate has high affinity for CAIX (Fig. 2b) and no aggregation was observed by size exclusion HPLC over a period of 7 days when samples were stored at either 0, -20, 4–25 °C (Figure S1). The reaction time required for conjugation of DFOSq to the antibody can be reduced from 18 h to 4 h by warming of the reaction mixture to 37 ^o^C and the use of a higher concentration of antibody (6 mg/mL). The comparatively easy synthesis of DFOSq-OEt, when compared to DFO-N-Suc-TFP, as well as the facile bioconjugations between DFOSq-OEt and girentixumab could be adventitious when preparing relatively large quantities of conjugate to support multi-center clinical trials.

Addition of [^89^Zr]Zr^IV^ (1 M oxalic acid) that had been neutralized with Na_2_CO_3_ (1 M) to a mixture of DFOSq-girentuximab in HEPES buffer allowed isolation of [^89^Zr]ZrDFOSq-girentuximab (radiochemical yield of ~ 55%, 500 MBq/mg). Evaluation of [^89^Zr]ZrDFOSq-girentuximab in high CAIX expressing HT29 tumour-bearing mouse model showed high tumour uptake and low background which was confirmed by a biodistribution study where the tumour uptake was 43.6 ± 2.6 IA/g at 144 h post injection.

Encouraged by the promising results with [^89^Zr]ZrDFOSq-girentuximab in this mouse model and promising clinical trials investigating [^89^Zr]ZrDFO-N-suc-girentuximab as a diagnostic tool, we developed an automated radiochemical synthesis. The first step of the automated synthesis was to prepare [^89^Zr]ZrCl_4_ (Noor et al. 2024). A commercial iPHASE MultiSyn synthesis module based on disposable cassettes equipped with an ion exchange cartridge in the bicarbonate form (PS-HCO_3_ SAX) is used to efficiently trap [^89^Zr]Zr^IV^ from a mixture of the radionuclide in oxalic acid. Elution with a mixture of HCl (0.1 M) in NaCl (1.0 M) allows elution of a species that presumably approximates [^89^Zr]ZrCl_4_. This mixture can be used for direct radiolabeling of DFOSq-girentuximab in aqueous sodium acetate (0.25 M) at room temperature to give [^89^Zr]ZrDFOSq-girentuximab with ~ 93% radiochemical yield (> 95% radiochemical purity and specific activities of 500 MBq/mg were easily achieved) without the need for further purification by size-exclusion/PD-10 chromatography. The lower concentration of acid used in the approach presented here allows the pH to be buffered with sodium acetate rather than requiring neutralization with a stronger base such as sodium carbonate. This is an advantage as, in some instances, we have found the use of sodium carbonate to neutralise [^89^Zr]Zr^IV^ mixtures in oxalic acid to be unpredictable leading to the formation of radioactive precipitates that we assumed to be due to the formation of zirconium hydroxide and colloidal species.

## Conclusions

The monoclonal antibody girentuximab, that binds to CAIX, was conjugated to DFOSq and the modified monoclonal antibody was radiolabelled with zirconium-89 using [^89^Zr][Zr(ox)_4_]^4−^ as precursor to give [^89^Zr]ZrDFOSq-girentuximab. This tracer displayed good tumour uptake and produced good quality PET images in a HT29 xenograft model. An automated process for synthesis of [^89^Zr]ZrDFOSq-girentuximab was developed that used [^89^Zr]ZrCl_4_ as a precursor. Advantages of this new automated approach for the synthesis of [^89^Zr]ZrDFOSq-girentuximab include: Removal of the requirement to use a size-exclusion filtration, early removal of potentially toxic oxalate/oxalic acid, isolation of the product in higher radiochemical yield whilst reducing radiation exposure to operators producing the tracer. The methodology to add DFOSq to antibodies, that is presented here, and the automated synthesis for radiolabeling the resulting conjugate with zirconioum-89 could be readily adapted to other antibodies.

## Methods

### General procedures

Unless otherwise stated, all reagents were purchased from commercial sources and used without further purification. Mass spectrometry (MS) was performed using Mass Hunter version B.06.00 with BioConfirm software using the maximum entropy protein deconvolution algorithm. The chelator to antibody ratio was estimated from the peak abundances. Size exclusion chromatography of cold antibody conjugates were performed on an Agilent 1100 Series HPLC system. A Phenomenex Yarra 3000, 3 μm 4.6 × 300 mm column was used with a flow rate of 0.35 mL/min of phosphate buffer (0.1 M, pH 6.8) as eluent. Approximately 10–20 µg of antibody samples were loaded onto the column and detected at wavelengths of λ = 214, 230, 254, 280 nm and 320 nm. Analysis of radioactive samples using the same size exclusion column as above on a Shimadzu SCL-10 A VP/LC-10 AT VP system with a Shimadzu SPD-10 A VP UV detector followed by a radiation detector (Ortec model 276 photomultiplier base with preamplifier, Ortec 925- SCINT ACE mate preamplifier, BIAS supply and SCA, Bicron 1 M 11/2 photomultiplier tube). Typically approximately 2–4 µg (1–2 MBq) of protein sample is loaded onto the column. Crude radiolabelled products were purified on a PD-10 size exclusion column (Sephadex G-25, GE healthcare, Cat# 17085101) using 0.5% sodium gentisate in PBS (20 mM, pH 7.4) as eluent.

### Synthesis of DFOSq-girentuximab

To a solution of girentuximab antibody (1 equivalent, 750 µg) in borate buffer (pH 9.0, 0.1 M) was added DFOSq (40 equivalents, 138 µg in dimethyl sulfoxide) at room temperature to give a final concentration of 1 mg/mL of the reaction mixture. The reaction mixture was incubated at room temperature for 18 h then filtered using Amicon^®^ 100 kDa centrifugal filters. The crude product was washed on the filter with HEPES buffer (10 mM, pH 7.4) and the concentrate collected and diluted with HEPES buffer to give DFOSq-girentuximab (1 mg/mL). The product was analysed by size exclusion chromatography and LC-MS which indicated a mixture of girentuximab with 1–6 chelators with an average 3.1 chelators per antibody.

### Synthesis of [^89^Zr]ZrDFOSq-girentuximab

[^89^Zr]Zr^IV^ in 1 M oxalic acid (123 µL, 110 MBq, Perkin Elmer NEZ308000MC) was diluted with MilliQ water (123 µL) to avoid precipitation and then was neutralized (pH 6–7) with a series of additions of aqueous Na_2_CO_3_ (1 M, 2 × 25 µL and 3 × 12 µL). HEPES buffer (0.5 M, pH 7.0, 116 µL) was then added to get a final solution of 0.25 M HEPES and the solution allowed to stand for 5 min before pH was tested again. After confirming a neutral pH, a solution of DFOSq-girentuximab (avg. 3:1 DFOsq/mAB, 110 µg) in HEPES buffer (0.1 M, 110 µL) was added and the reaction mixture was incubated at room temperature. The progress of the reaction was monitored by radio size exclusion-HPLC and after 40 min reaction time an aliquot was analysed and found to have a radiochemical yield of < 50%. Another 110 µg of antibody was added and the reaction mixture was left to stand for a further 30 min to achieve > 50% radiochemical yield. The crude [^89^Zr]Zr]DFOSq-girentuximab was purified on a PD-10 size exclusion column (Sephadex G-25, GE healthcare, Cat# 17085101) using 0.5% sodium gentisate in PBS (20 mM, pH 7.4) as eluent. The flow through was discarded and three fractions (3 × 0.5 mL) were collected containing 17.7, 33.6 and 7.7 MBq. The first two fractions were combined and tested for radiochemical purity by size exclusion-HPLC and diluted with 0.5% sodium gentisate in PBS (20 mM, pH 7.4) to a final volume of 1.35 mL containing approx. 51 MBq. Six doses (100 µL, 3.3 MBq each) of [^89^Zr]ZrDFOSq-girentuximab in PBS with 0.5% sodium gentisate (prefiltered for injection using a Millipore Millex^®^-HV 0.22 μm sterile filter unit) were prepared and administered to mice via tail vein injection.

### PET imaging and biodistribution

Female BALB/c nude mice (ARC, Western Australia, age: 8 weeks) were inoculated subcutaneously on the right flank with 5 × 10^6^ HT29 or 3.5 × 10^6^ MDA-MB-231 cells in 50% Matrigel. Mice were injected intravenously with [^89^Zr]ZrDFOSq-girentuximab (2–3 MBq, 100–130 µL). A cohort of animals comprising one MDA-MB-231 and three HT29 tumour bearing mice was anaesthetised using isoflurane. A CT image and 10 min static PET scans were acquired (Perkin Elmer, Sofie BioSciences Small Animal G8 PET/CT scanner). A cohort of three animals was euthanised at 24, 48 h post injection and after imaging at 144 h and selected tissues were removed, weighed and counted in a gamma counter. Tracer uptake was calculated as % injected activity/gram tissue (%ID/g). Statistical analysis of the data was performed using GraphPad Prism 7.04.

### Automated production of [^89^Zr]ZrDFOSq-girentuximab

The manufacturing room was cleaned in accordance with facility standard operating procedures and the proper Environmental Monitoring (EM) was performed and conformed to standard specification. The reagents were either sourced commercially or freshly prepared in the lab, 0.1 M HCl solution in 1 M sodium chloride was drawn in a sterile 1 mL syringe provided with the disposable cassette and stored capped with sterile needle until required. A volume of precursor solution corresponding to the mass of precursor required for the radiolabeling was added to a 1 mL 0.25 M sodium acetate vial followed by adding Na-gentisate (2.5%). The disposable cassette MSH-3000 was modified and assembled as shown in Fig. 5a then mounted on MultiSyn module Fig. 5b. The iPHASE Multisyn module was then switched on and the sequence scheme loaded and prompts followed to perform initial leak checks. Following the sequence prompts, the 1 mL syringe containing 0.1 M hydrochloric acid in 1 M sodium chloride solution was mounted at position 1 (manifold 1), the vial containing 10 mL saline for injection was placed at position 11 (manifold 4), the 1 mL 10x PBS syringe was placed at position 10 (manifold 4), a 100 mL water for injection bag was placed on a spiked tube connected to position 5 (manifold 2), the precursor solution was loaded via the center port into the reactor, the pre-prepared 15 mL sterile product collection vial in a shielded transport container was connected to the product delivery line at position 12 (manifold 4), and finally the [^89^Zr]Zr-oxalate in oxalic acid was connected to the module using a PEEK needle inlet at position 2 (manifold 1). The process was completed in ~ 45 min and the product collection vial removed. Final product volume was calculated (12–15 mL), the product activity recorded using a calibrated dose calibrator to calculate yield radiochemical yield (80–92%), and the product tested for QC analysis by radio-HPLC (95%), radio-TLC (> 95%), pH (Verhoeff et al. 2019, 2023; Merkx et al. 2021) and visual appearance parameters (colorless no visible particles). Analysis by iTLC confirmed the product was found to be stable up to 6 days EOS when stored at room temperature at an activity level of 886 MBq.

## Electronic supplementary material

Below is the link to the electronic supplementary material.

Supplementary Material 1

## Data Availability

All data generated or analysed during this study are included in this published article [and its Additional file 1].
