# Supplementary material for: Preclinical evaluation and automated synthesis of [89Zr]ZrDFOSquaramide-girentuximab for diagnostic imaging of carbonic anhydrase IX positive tumours
Source: EJNMMI Radiopharm Chem. 2024 Nov 26;9:80. doi: 10.1186/s41181-024-00310-x (PMC11599670; doi:10.1186/s41181-024-00310-x)
Supplement: Supplementary file 1 — Supplementary Material 1 [file 41181_2024_310_MOESM1_ESM.docx]

**Supplementary Material**

**Preclinical evaluation and automated synthesis of [^89^Zr]ZrDFOSquaramide-girentuximab for diagnostic imaging of carbonic anhydrase IX positive tumours**

Asif Noor,^1*^ Emily R. McGowan,^1^ Jessica K. Van Zuylekom,^2^ Carleen Cullinane,^2,3†^ Peter D. Roselt,^3,6^ Rodney J. Hicks^2,3,4^ Michael P. Wheatcroft,^5^ and Paul S. Donnelly^1^*

^1^School of Chemistry and Bio21 Molecular Science and Biotechnology Institute University of Melbourne, Parkville, Victoria 3010, Australia.

^2^Research Division, Peter MacCallum Cancer Centre, Melbourne, Victoria 3000, Australia.

^3^Sir Peter MacCallum Department of Oncology, The University of Melbourne, Parkville, VIC, Australia.

^4^Melbourne Theranostic Innovation Centre, Level 8/14-20 Blackwood St, North Melbourne VIC 3051, Australia

^5^Telix Pharmaceuticals Limited, Suite 401, 55 Flemington Road, North Melbourne, Victoria 3051, Australia.

^6^Department of Radiopharmaceutical Sciences, Cancer Imaging, Peter MacCallum Cancer Centre, Melbourne, Victoria 3000, Australia

^†^ Deceased.

Correspondence: [asif.noor@unimelb.edu.au](mailto:asif.noor@unimelb.edu.au); [pauld@unimelb.edu.au](mailto:pauld@unimelb.edu.au)

Table of Contents

[Synthesis of DFOSq-Girentuximab 3](#_Toc181779630)

[Selected SEC-HPLC Traces 3](#_Toc181779631)

[MicroPET Imaging 4](#_Toc181779632)

[Biodistribution Analysis 5](#_Toc181779633)

[Automated synthesis 5](#_Toc181779634)

[Stability of [^89^Zr]ZrDFOSq-girentuximab in human serum 6](#_Toc181779635)

# **Synthesis of DFOSq-Girentuximab**

**Table S1** Bioconjugation of DFOSq with Girentuximab

| DFO-Sq Equivalents | Reaction  Time (h) | Reaction Concentration (mg/ml) | No of Chelators attached | Average chelator to antibody ratio (chelator/antibody) |
| --- | --- | --- | --- | --- |
| 20 | **18** | **1** | **0-4** | **1.3** |
| 40 | **18** | **1** | **1-6** | **3.3** |
| 60 | **18** | **1** | **2-8** | **5.3** |
| 80 | **18** | **1** | **5-11** | **8.0** |
| 40* | **4** | **6** | **0-6** | **3.0** |

*Reaction performed at 37 °C

# **Selected SEC-HPLC Traces**

**Figure S1** size exclusion chromatogram (SEC) traces for the DFOsq-girentuximab sample incubated at different temperature of time over 7 days

# **MicroPET Imaging**


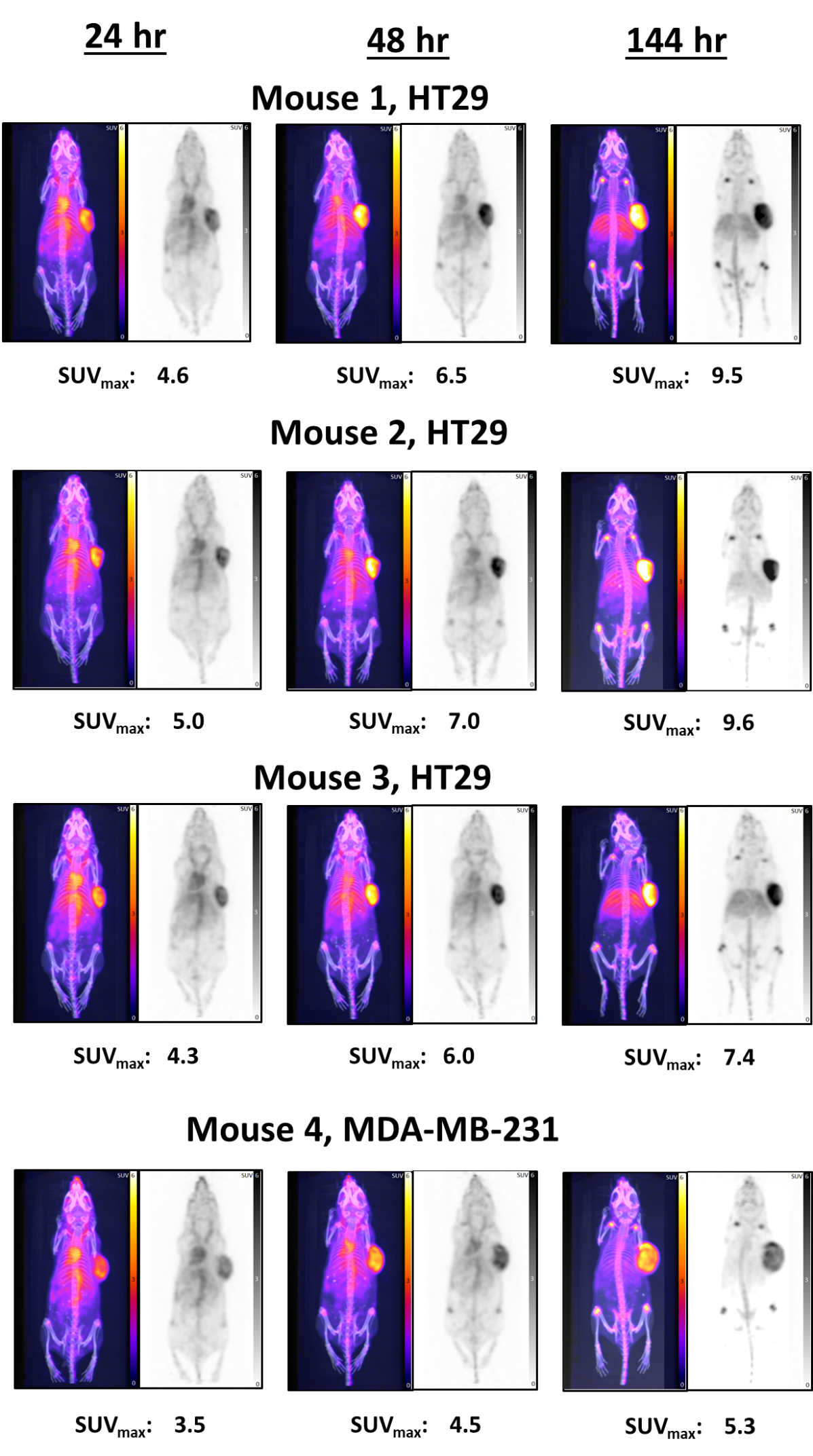


**Figure S2** Whole-body microPET maximum intensity projection (MIP) and CT images of mice bearing xenograft HT29 and MDA-MB-231 tumors after 24, 48 and 144 h post injection of ^89^ZrDFOSq-girentuximab

# **Biodistribution Analysis**

**Figure S3** Ex-vivo biodistribution analysis. Mice was euthanised at 24, 48 and 144 hr after injection with ^89^ZrDFO-Sq-girentuximab in MDA-MB-231. Tissues were harvested, weighed and counted on a gamma counter. Tracer uptake is expressed as percent injected dose/gram tissue. Data represents n = 1

# **Automated synthesis**

**Figure S4.** Representative example of Radio-SEC of [^89^Zr]ZrDFOSq-girentuximab synthesized on automated process

**Figure S5.** Representative example of Radio-TLC [^89^Zr]Zr-DFOSq-girentuximab (left) synthesized on automated process and buffered ^89^Zr (right) in 0.1M citrate buffer.

# **Stability of [^89^Zr]ZrDFOSq-girentuximab in human serum**

[^89^Zr]ZrDFOSq-girentuximab (30 MBq, 30 µg) was added to human serum (100 µL, Sigma Aldrich, human male AB plasma, USA origin, sterile-filtered). The sample was incubated at 37 °C and the mixture was analyzed after 1, 4, 24 and 48 hours by instant thin layer chromatography (mobile phase citrate 20 mM). [^89^Zr]ZrDFOSq-girentuximab was stable for 48 hours (Figure S7).


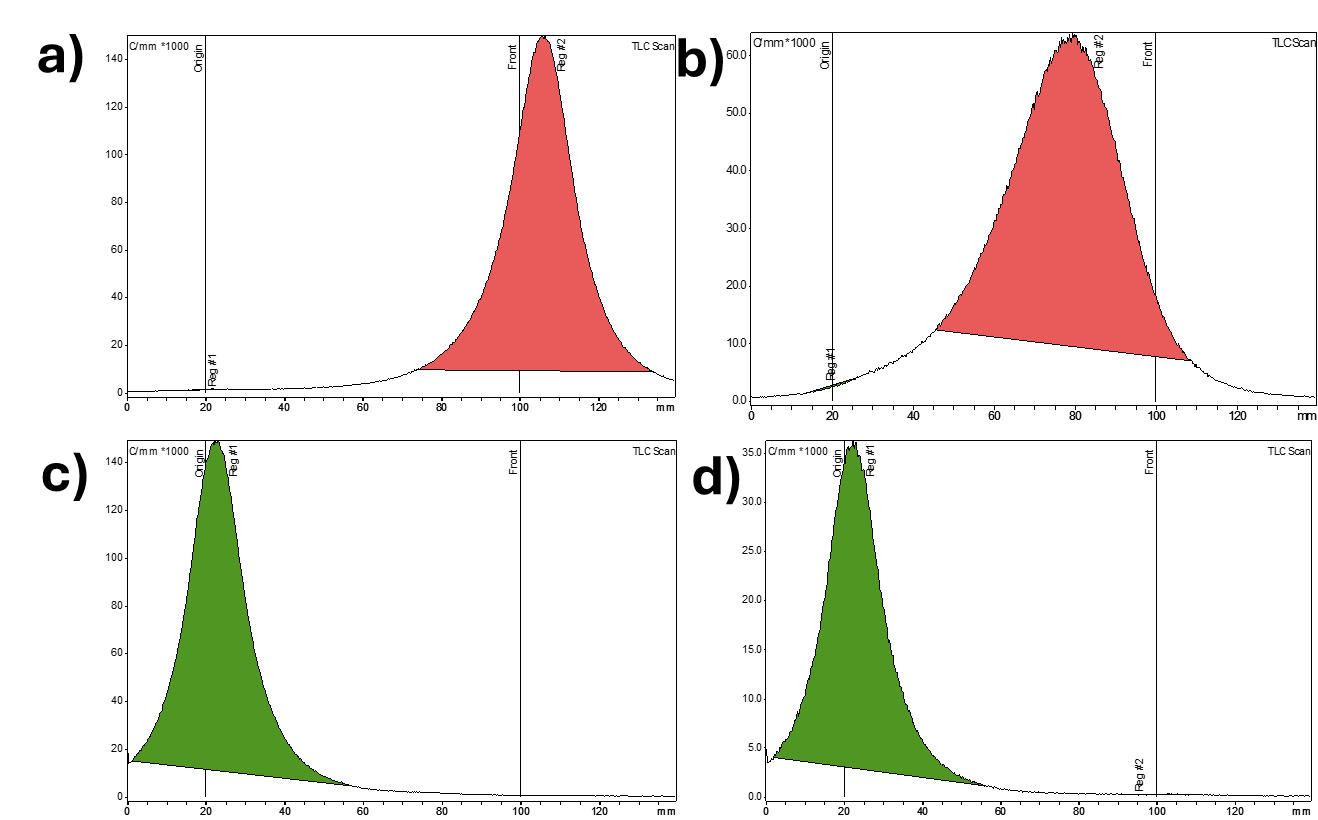


**Figure S6.** Radio-TLC chromatograms of: a) [^89^Zr][Zr(ox)_4_]^4-^_;_ b) [^89^Zr][Zr-DFOSq-OH]; c) [^89^Zr]ZrDFOSq-girentuximab; d) [^89^Zr]ZrDFOSq-girentuximab after 48 hours incubation in human serum at 37 °C.

**Figure S7.** Radio-TLC [^89^Zr]ZrDFOSq-girentuximab (886 MBq) synthesized by automated process after 6 days post production, stored at RT.
